# Inhibitory Fc-Gamma IIb Receptor Signaling Induced by Multivalent IgG-Fc Is Dependent on Sialylation

**DOI:** 10.3390/cells12172130

**Published:** 2023-08-23

**Authors:** Christopher Beneduce, Stephanie Nguyen, Nathaniel Washburn, John Schaeck, Robin Meccariello, Kimberly Holte, Daniel Ortiz, Anthony M. Manning, Carlos J. Bosques, Elma Kurtagic

**Affiliations:** 1 Momenta Pharmaceuticals Inc., Cambridge, MA 02142, USA; 2 Janssen Research & Development, Cambridge, MA 02142, USA

**Keywords:** immunoglobulin Fc, sialylation, therapeutics, FcγRIIb

## Abstract

Immunoglobulin (IgG) Fc glycosylation has been shown to be important for the biological activity of antibodies. Fc sialylation is important for the anti-inflammatory activity of IgGs. However, evaluating the structure–activity relationship (SAR) of antibody Fc glycosylation has been hindered using simplified in vitro models in which antibodies are often displayed in monomeric forms. Presenting antibodies in monomeric forms may not accurately replicate the natural environment of the antibodies when binding their antigen in vivo. To address these limitations, we used different Fc-containing molecules, displaying their Fc domains in monovalent and multivalent fashion. Given the inhibitory role of Fc gamma receptor IIb (FcγRIIb) in autoimmune and inflammatory diseases, we focused on evaluating the impact of Fc sialylation on the activation of FcγRIIb. We report for the first time that in human cellular systems, sialic acid mediates the induction of FcγRIIb phosphorylation by IgG-Fc when the IgG-Fc is displayed in a multivalent fashion. This effect was observed with different types of therapeutic agents such as sialylated anti-TNFα antibodies, sialylated IVIg and sialylated recombinant multivalent Fc products. These studies represent the first report of the specific effects of Fc sialylation on FcγRIIb signaling on human immune cells and may help in the characterization of the anti-inflammatory activity of Fc-containing therapeutic candidates.

## 1. Introduction

Antibodies have a well-defined role in the pathogenesis of many autoimmune disorders but also in mediating anti-inflammatory activities. Antibodies of the IgG isotype interact with Fc gamma receptors (FcγRs) expressed widely on many immune cells. Antibodies can bind their cognate antigen via the variable fab domains and exert their activity by binding to the FcγRs via their Fc domain, initiating a broad spectrum of effector functions important in host defense [1,2]. Several activating Fc gamma receptors are expressed in human cells, including FcγRI, IIa, IIc and IIIa, as well as the only inhibitory FcγRIIb, through which IgGs can exert activity. The activating Fc gamma receptors signal via a highly conserved immunoreceptor tyrosine-based activation motif (ITAM). In contrast, FcγRIIb contains an immunoreceptor tyrosine-based inhibition motif (ITIM) in its a-chain. The inhibitory FcγRIIb is typically expressed together with activating Fc receptors in many immune cells. One notable exception is B cells as they express only FcγRIIb, with no expression of activating Fc gamma receptors. With the exception of FcγR1, FcγRs bind monomeric IgGs with low affinity and typically require IgGs to be presented in a multivalent format, as is the case upon interacting with their cognate antigens, resulting in the formation of immune complexes (ICs). Subsequently, ICs can trigger inflammatory processes through activating FcγRs, or they can quell their pro-inflammatory activities through activation of the inhibitory FcγRIIb.

However, the pro and anti-inflammatory activities of IgGs are not only modulated by their subclass. Their Fc glycosylation and valency play a major role. The Fc domain is heterogeneous and, in addition to the four distinct subclasses of human IgG (IgG1, IgG2, IgG3, and IgG4), the Fc domain exhibits diversity in its glycosylation states. IgG glycosylation is known to be crucial in modulating the in vivo activity of IgGs. All IgG molecules contain a single conserved N-linked glycosylation site (Asn-297) in each of the constant heavy 2 (CH2) domains, critical for maintaining their pro-inflammatory and anti-inflammatory effector functions [3,4]. The sugar moiety attached to Asp-297 predominantly consists of an octasaccharide biantennary structure, comprising four N-acetylglucosamines (GlcNac), three mannoses and one fucose, which may contain terminal galactose or sialic acid residues. These glycans help maintain the quaternary structure and the stability of the Fc [5,6], and are vitally involved in all FcγR binding [7,8]. Evidence that deglycosylated IgG antibodies are unable to mediate in vivo-triggered inflammatory responses accounts for the requirement of the glycans for FcγR binding [9]. For instance, sialylated IgG glycoforms can adversely impact IgG-Fc pro-inflammatory function [10], whilst fucosylation has been shown to be a sensitive modulator of antibody-dependent cellular cytotoxicity. It has been previously shown that the anti-inflammatory activity of intravenous immunoglobulins (IVIg) is dependent on Fc sialylation [11,12]. It was also demonstrated that increasing the levels of Fc sialylation can increase the anti-inflammatory potency of IVIg in animal models [11,13]. The association between the FcγRIIb receptor and the anti-inflammatory activity of IVIg has been documented in human patients with chronic inflammatory demyelinating polyneuropathy (CIDP) [14]. Although the precise mechanisms of action for the Fc sialyation-associated anti-inflammatory activity of IVIg remain a subject of debate, previous studies point to the inhibitory FcγRIIb receptor as a key factor [12,15,16,17,18,19]. IVIg can potentially bind to soluble or cell surface antigens [20,21,22,23], and antibody specificities within IVIg [24,25,26,27,28] may contribute towards the anti-inflammatory activities of IVIg. Even though the mechanism by which sialylated IgGs exert their anti-inflammatory activities has been explored for years, there is still very little understanding of the interplay between Fc valency, sialylation, FcγR binding and activation of the downstream signals in human cellular systems. This is in part due to the simplified in vitro models employing monovalent IgGs to simulate activity. Presenting antibodies in the monomeric forms may suffer from significant limitations since these conditions may not accurately replicate the natural environment or properties of the antibodies in vivo. For example, Fc portions of antibodies may not be displayed in multivalent formats as when antibodies encounter their cognate antigens in nature (i.e., opsonization of bacterial particles, binding of soluble antigens, binding of cell surface receptors). In fact, it was recently reported that monomeric IgG–FcγR interaction analysis did not predict IgG activity [29]. On the other hand, the size of the multivalent IgGs (i.e., immune complex generated upon antibody binding to its target antigen) can mitigate the requirement for IgG glycosylation in vitro [30] further adding to the complexity of the FcγR system.

Given the clinical relevance of the inhibitory FcγRIIb receptor in inflammatory diseases and the previous association with Fc sialylation-dependent anti-inflammatory activity, in this study we evaluated the in vitro effects of Fc sialylation on FcγRIIb modulation in human cellular systems. Specifically, to simulate the natural environment of antibodies when binding their antigens, we focused on assays displaying different Fc molecules in multivalent forms. To do so, we employed the therapeutic antibodies adalimumab and IVIg that were subjected to site-specific Fc sialylation as well as Fc molecules of discrete valencies. Our results demonstrate that in human cellular systems, multivalent Fcs led to robust FcγRIIb phosphorylation in a sialic acid-dependent fashion. To our knowledge, this is the first study to demonstrate the specific effects of Fc sialylation on FcγRIIb modulation in human cellular systems. Therefore, our study improves the understanding of the interplay between Fc valency, glycosylation and FcγRIIb activation, which may be beneficial for the treatment of autoimmune diseases.

## 2. Materials and Methods

### 2.1. Generation of Tetra-Sialylated IVIg and Desialylated IVIg

The Fc N-glycans of IVIg (Privigen; CSL Behring, King of Prussia, PA, USA) were galactosylated with B4GalT1 and UDP-gal (Roche, Indianapolis, IN, USA) and subsequently sialylated with ST6Gal1 and CMP-NANA (Roche, Indianapolis, IN, USA). After multi-column purification to remove the enzymes and nucleotide sugars, the material was formulated into 250 mM glycine at pH 5.0. The level of N-glycan hypersialylation was determined by LC-MS/MS glycopeptide analysis. Desialylated IVIg was obtained by treating IVIg (Privige; CSL Behring, King of Prussia, PA, USA) in its formulation buffer (250 mM glycine at pH 5.0) with a mixture of neuraminidases (neuraminidase *Arthrobacter ureafaciens*, neuraminidase *Vibrio cholera*, neuraminidase *Clostridium perfingens*, all from Roche) for 72 h at 37 °C. The material was then purified using Protein G (HiTrap Protein G HP; GE Healthcare, Boston, MA, USA) and then formulated into 1× PBS. The level of desialylation was determined by LC-MS/MS glycopeptide analysis.

### 2.2. Generation of Multivalent Fc Molecules

The Fc3Y, Fc5X, and Fc5Y molecules were generated as described in Ortiz, 2016. Briefly, the molecules of discrete sizes and configurations were engineered using mutations based on the knobs-into-holes technology for heterodimerization and electrostatic steering for homodimerization. Placement of the homodimerization and heterodimerization Fc domains within the constructs coupled with regulated expression of the precursor peptides using separate plasmids enabled controlled assembly of Fc oligomers of different sizes and configurations. For flow cytometry analyses, constructs were labeled with VivoTag 645 (Perkin Elmer, Waltham, MA, USA) following the manufacturer’s instructions. The labeled constructs were purified by Size Exclusion Chromatography (SEC), desalted, and stored in PBS as described below. Dye loading was calculated following the manufacturer’s instructions, using extinction coefficients for the Fc3Y constructs (e~215,000 M^−^^1^cm^−^^1^) and the VivoTag 645 Dye (e 210,000 M^−^^1^cm^−^^1^).

### 2.3. Fc Glycosylation Analyses by Glycopeptide Liquid Chromatography–Mass Spectrometry (LC-MS/MS)

The glycosylation of the Fc domain of each of the immunoglobulin test articles was characterized using peptide LC-MS/MS after tryptic digestion as described previously [13]. In short, 50 µg of each of the proteins was reduced with dithiothreitol (10 mM) in 6 M guanidine HCl for 30 min at 65 °C. The free cysteine residues were alkylated with iodoacetamide (30 mM) at room temperature for 1 h. The reduced and alkylated protein was dialyzed across a 10,000 Da molecular weight cut-off filter into 4 L of 25 mM ammonium bicarbonate overnight at 4 °C. After dialysis, trypsin (Promega, Madison, WI, USA) was added to the sample for proteolysis, which was carried out in a Barocycler (NEP 2320; Pressure Biosciences, Inc., South Easton, MA, USA). The pressure was cycled between 20,000 psi and ambient pressure at 37 °C for a total of 30 cycles in 1 h. LC-MS/MS analysis of the tryptic digests was performed on an Ultimate 3000 (Thermo Fisher Scientific, Waltham, MA, USA) RSLC system and a QExactive (Thermo Fisher Scientific, Waltham, MA, USA) Mass Spectrometer. Peptides were separated on a BEH PepMap (Waters) 1 × 100 mm HPLC Column using 0.1% FA in water and 0.1% FA in acetonitrile as the mobile phases at a flow rate of 75 µL/min. Glycosylation was quantified for each of the test articles based on the extracted ion area for each glycoform using the predicted tryptic peptide for IgG1 (EEQYNSTYR). Glycosylation analysis of IVIg, which contains each of the 4 subclasses, was restricted to the IgG1 subclass as this makes up >60% of the pool, and previous analyses have shown that enzymatic sialylation was not influenced by subclass (Washburn et al. 2015). The relative abundance was calculated using the extracted ion area of the two most abundant charge states (z = 3 and z = 2) for each glycopeptide.

### 2.4. Measuring Complex Formation of TNFα-(S2) Anti-TNFα

Recombinant, monoclonal anti-TNFα antibody (prepared in house) and soluble, trimeric TNFα (R&D Systems, Minneapolis, MN, USA) were mixed in a 3:1 molar ratio to form complexes composed of three anti-TNFα molecules binding one TNFα trimer. The mixtures were incubated for 24 or 48 h at 37 °C. The average molecular weights of the TNFα immune complexes were measured using a Size Exclusion Chromatography (SEC) system coupled with Multi-Angle Light Scattering (MALS) and Refractive Index (RI) detection. HPLC data were collected on an Agilent 1100 liquid chromatograph (Palo Alto, CA, USA) equipped with an OptiLab refractive index detector (Wyatt Technology, Santa Barbara, CA, USA) and a MiniDawn TREOS light scattering detector (Wyatt Technology, Santa Barbara, CA, USA). A Bio SEC-5 (Agilent, Palo Alto, CA, USA) size exclusion column (4.6 × 300 mm, 5 μm) was used for the analysis. An isocratic mobile phase of 0.15 M sodium phosphate (pH 7.0) at a flow rate of 0.35 mL/min was employed for the separation at an ambient column temperature. An injection volume of 25 µL was employed. Data were processed using both Empower 2 software (Build 2154, Waters, Milford, MA, USA) and ASTRA software (Version 5.3.4, Wyatt Technology, Santa Barbara, CA, USA). Each sample (monomer or complex) was analyzed directly post sample preparation.

### 2.5. Cell-Based FcγR Binding Measurements

The relative binding of monovalent and multivalent samples to FcγRs was measured using cell-based homogeneous time-resolved fluorescence competition assays (TR-FRET CisBio assays) according to manufacturer’s instructions. The kits used contained HEK cells expressing CD16aV158, CD32aH131, CD32b, and CD64. A 10-point, 3-fold serial dilution series, plus one blank per sample, was generated using an automated liquid handler (Freedom EVOware 150; Tecan, Mannedorf, CH). Assay plates were read on a PHERAstar fluorescent reader (BMG Labtech GmbH, Ortenberg, DE) at 665 and 620 nm. GraphPad Prism 7 software was used to calculate IC50 values for each sample.

### 2.6. Staphylococcus aureus Bioparticle Coating with IVIg

An *S. aureus* (Thermo Fisher Scientific, Waltham, MA, USA) vial was resuspended in 5 mL PBS. The vial was vortexed 3 × 15 s at the highest setting. The bioparticle solution was combined with IVIg, S2-IVIg or ds-IVIg in a 1:1 ratio. The mixture was allowed to rotate for 1 h at 37 °C. Subsequently, each tube was centrifuged at 1500× *g* for 10 min at 4 °C; the supernatants were removed and the pellet was resuspended in 1 mL PBS. The centrifugation and washing steps were repeated for a total of two washes with PBS. Finally, the washed pellet was resuspended in 200 µL of assay media.

### 2.7. Cell Treatments and Immunoblot Detection of Phosphorylated FcγRIIb in Daudi B Cell Lines

Daudi B cells (ATCC_CCL-213) were plated at 3 × 10^6^ cells/well in a 24-well plate. The cells were stimulated with respective complexes for 30 min at 37 °C (see details in figure legends). Subsequently, the cells were washed 2× with the ice-cold PBS and lysed in Meso Scale Discovery (MSD) lysis buffer supplemented with protease and phosphatase inhibitors. The samples were resolved on 4–12% SDS-PAGE (Thermo Fisher Scientific, Waltham, MA, USA) and transferred onto nitrocellulose membranes. WB was performed to detect phospho Y292 FcγRIIb (ab68423; Abcam, Waltham, MA, USA), total Akt (Cell Signaling Technologies, Danvers, MA, USA) or β-actin (Cell Signaling Technologies, Danvers, MA, USA). Densitometry measurements were performed with an Odyssey CLx (LI-COR, Lincoln, NE, USA) device. The raw fluorescent units of phospho targets were divided by the total target to obtain relative expression values for each signal. Fold changes with each treatment compared with the control were calculated.

To generate peripheral blood mononuclear cells (PBMCs) for treatments, whole blood was drawn from healthy donors into EDTA Vaccutainer Tubes (BD, Franklin Lakes, NJ, USA) after informed consent was obtained. The blood was diluted 1:1 with PBS and PBMCs were isolated by density centrifugation on Histopaque 1077 (Sigma Corporation, St. Louis, MO, USA). The PBMCs were washed twice in PBS with 0.5 mM EDTA and 0.5% BSA. After washing, the cells were resuspended in RPMI1640 with 10% Ultra Low IgG FBS (Thermo Fisher Scientific, Waltham, MA, USA). The PBMCs were stimulated as described above for the Daudi cells.

### 2.8. Flow Cytometry Analysis of Human PBMCs

PBMCs were isolated as described above. Approximately 0.75 × 10^6^ PBMCs in staining buffer (PBS, 2% heat-inactivated fetal bovine serum, 1 mM EDTA) were seeded into a 96-well V-Bottom plate. The PBMCs were stained for 45 min at 4 °C with a pre-titrated antibody panel to identify major PBMC subsets (the panel description and gating strategy can be found in Supplementary Figure S5). The cells were simultaneously stained with the VivoTag645-labeled Fc constructs or an APC-labeled Anti-FcγRIIb/c antibody (Clone 4F5; BioLegend, San Diego, CA, USA). Fluorescence-Minus-One (FMO) and isotype controls were included in each experiment. Brilliant Violet Staining Buffer (BD, Franklin Lakes, NJ, USA) was included in each staining reaction. After staining, the cells were washed twice with staining buffer and fixed in 1% PFA, prepared in PBS, for 20 min at room temperature. The cells were washed once more and resuspended in 200 mL of staining buffer. Then, ~200,000 single cell events were acquired on a Cytek Aurora flow cytometer. The raw data were unmixed using SpectroFlo (Version 2.5, Cytek Biosciences, Fremont, CA, USA) and was further analyzed using FlowJo (Version 10.7, BD, Franklin Lakes, NJ, USA). Fluorescence measurements are reported as the geometric mean fluorescence intensity (Geo. MFI).

### 2.9. Statistics

For all experiments involving Western blot densitometry or the Cisbio TR-FRET assay, significance was calculated using a Welch’s ANOVA followed by a Dunnet’s test of significance for multiple comparisons. Welch’s ANOVA was selected over traditional one-way ANOVA due to the non-homogeneity of variances in most experiments. For the flow cytometry experiments, significance was determined using multiple unpaired *t*-tests followed by a post hoc multiple comparison correction using the Holm–Sidak method.

## 3. Results

### 3.1. IVIg-Induced FcγRIIb Signaling in Human Immune Cells Is Dependent on Sialylation

**Figure 1:**
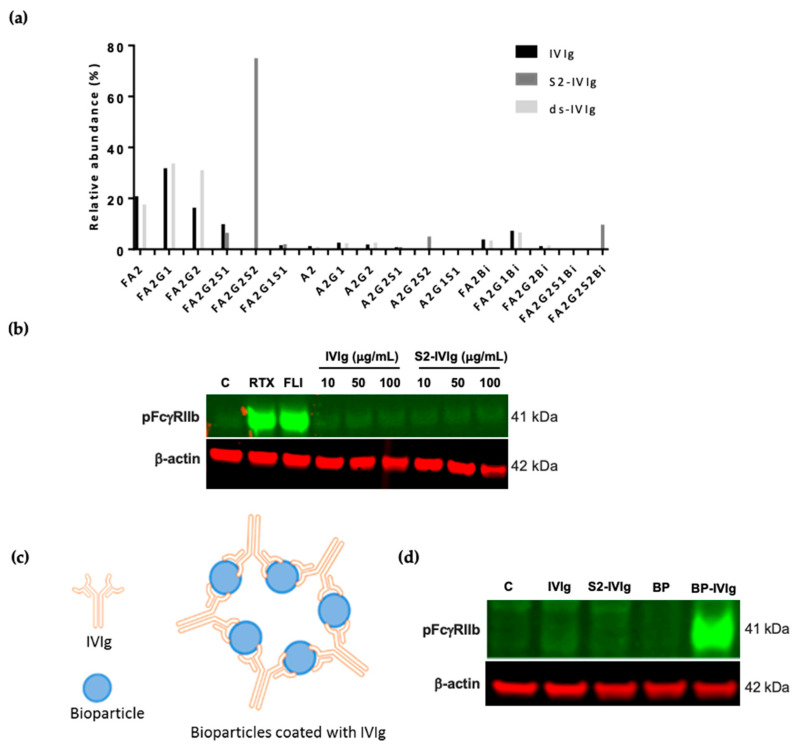
IVIg induces FcγRIIb signaling when presented in polymeric fashion. (**a**) IgG1 Fc N-glycopeptide profile for IVIg, S2-IVI and desialylated IVIg (ds-IVIg) measured using LC-MS/MS. Oxford nomenclature for N-linked glycans: all N-glycans have two core GlcNAcs; F at the start of the abbreviation = core fucose; Ax, number x = the number of antenna (GlcNAc) on trimannosyl core; Gx, x = number of linked galactose on antenna; Sx, x = number of sialic acids linked to galactose; Mx = high mannose x. (**b**) Western blot of phospho FcγRIIb (pFcγRIIb) and β-actin after 30 min stimulation of Daudi cells with rituximab (RTX) positive control, FcγRIIb agonistic antibody (FLI), and monomeric IVIg and S2-IVIg in solution; representative data shown from *n* > 6 independent observations. (**c**) Schematic of IVIg-coated *S. aureus* bioparticles. (**d**) Western blot of phospho FcγRIIb and β-actin after 30 min stimulation of Daudi cells with monomeric IVIg, S2-IVIg, bioparticles (BP) or IVIg-coated bioparticles; representative data from *n* > 6 independent observations. β-actin was used as a protein loading control.

The anti-inflammatory activity of intravenous immunoglobulins (IVIg) has been associated with Fc sialylation and the modulation of FcγRIIb. However previous studies have predominantly relied on rodent systems, and very limited data based on human cellular systems exist. It is noted in the literature that IVIg pools often contain approximately 5–15% Fc sialylation in the mixture, and it has been shown that this small sialylated IVIg fraction is critical for the anti-inflammatory activity of IVIg [11,16]. We subjected IVIg to IgG1 N-glycopeptide analysis, which demonstrated that ~20% of the Fc-glycans contained sialic acids (Figure 1a). Subsequently, it was evaluated whether the sialylation effects described above could be at play in mediating IVIg function. To do so, we focused on B cells, which exclusively express inhibitory FcγRIIb and contain no activating FcγRs. Additionally, FcγRIIb activation can be monitored by phosphorylation of its ITIM domain. Hence, this principle was used to probe the activity of IVIg on FcγRIIb signaling. Interestingly, IVIg presented in a monomeric format had no impact on FcγRIIb phosphorylation in Daudi cells (Figure 1b). Rituximab (RTX) induced potent FcγRIIb signaling, as previously reported [31], and was used as a positive control in this assay.

It has been proposed that therapeutic anti-inflammatory activity of IVIg is exerted in part through opsonization of soluble and cell antigens [20,21,22,23]. A study by Siragam et al. demonstrated that, like IVIg, antibodies to soluble antigens can ameliorate ITP in an FcγRIIB-dependent manner [32]. Furthermore, sialylation in the Fc region has been shown to contribute towards the anti-inflammatory activities of IVIg, mainly through the inhibitory receptor FcγRIIb. In addition, the size and valency of the IgG immune complexes strongly modulate Fc–receptor interactions. However, to interrogate this IVIg effect in vitro has been challenging as its Fc needs to be presented in a polymeric fashion. Previous attempts to address this issue have either used heat-aggregated IgG or multimeric IgG generated by co-incubation of IgG with anti-IgG F(ab’)2 fragments. Even though both approaches result in IgG aggregation, they may not reflect the interaction of IgG with its cognate antigen and do not allow for evaluation of the effect of the Fc chain interaction with FcγRs in a proper conformation. To resolve this issue, *S. aureus* bioparticles (BP) were opsonized with IVIg (Figure 1c) and tested in the B cell signaling assay. Remarkably, FcγRIIb was phosphorylated with BPs opsonized with IVIg, whereas no signaling was observed with monomeric IVIg (Figure 1d).

### 3.2. IVIg-Induced FcγRIIb Signaling Is Sensitive to Sialylation Levels

**Figure 2:**
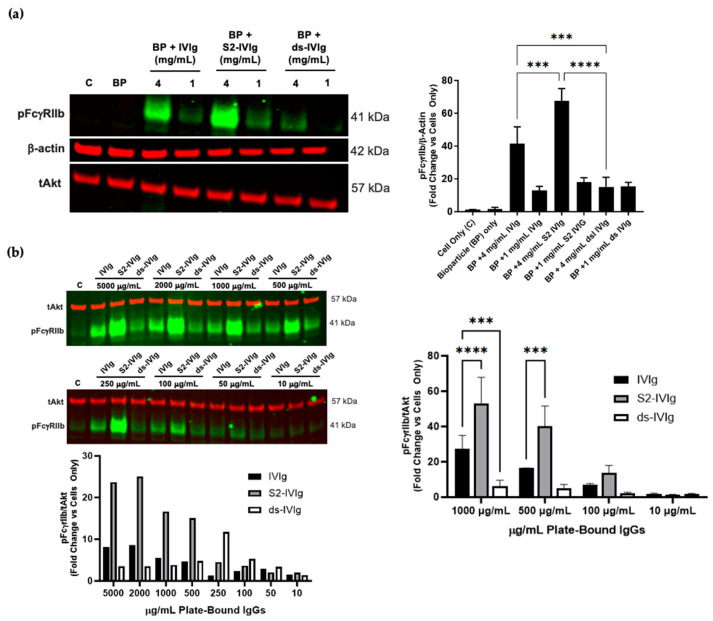
IVIg-induced FcγRIIb signaling is sialylation dependent. (**a**) Left: Western blot of phospho FcγRIIb, total Akt and β-actin after 30 min stimulation of Daudi cells with bioparticles (BP) alone or BPs coated with IVIg, S2-IVIg or desialylated IVIg (ds-IVIg); right: densitometry of compiled Western blots from *n* = 3 independent experiments. (**b**) Western blot of phospho FcγRIIb and total Akt (tAkt) after 30 min stimulation of Daudi cells with extended dose response of plate-bound IVIg, S2-IVIg or ds-IVIg. Lower panel: densitometry of the Western blot. Right panel: densitometry results of *n* = 3 independent experiments using shortened dose response. tAkt and β-actin served as loading controls. Description of statistical methods can be found in the methods section. *** *p* < 0.005, **** *p* < 0.0001.

Since IVIg is naturally sialylated, to gain a better understanding of the dependence of IVIg sialylation levels on the IVIg-induced FcγRIIb signaling, a desialylated IVIg devoid of sialic acids (ds-IVIg) and a tetra-Fc-sialylated IVIg were generated. IgG1 Fc N-glycopeptide analysis of IVIg and S2-IVIg demonstrated the presence of ~20% sialylated IVIg in the starting material and more than 90% enzymatically-introduced sialylation in S2-IVIg, whilst ds-IVIg contained no sialic acid (Figure 1a). IVIg and S2-IVIg-coated particles induced potent pFcγRIIb levels compared with the control at the highest concentration tested, with sialic acid-containing complexes being more potent. By contrast, bioparticles coated with desialylated IVIg were significantly less potent in inducing pFcγRIIb at either dose tested (Figure 2a).

Although FcγRIIb phosphorylation was triggered with IgG-coated bioparticles, it was apparent that opsonization of bioparticles with different IVIg preparations did not provide a wide dose–response range, most likely because the system cannot be well controlled. For better control of the IgG amounts and robustness of the assay, FcγRIIb signaling in Daudi cells was evaluated using specific quantities of immobilized S2-IVIg, IVIg and ds-IVIg on tissue culture plates. This procedure allowed for testing a range of concentrations of each molecule. The results in Figure 2b indicate that the IVIg-induced FcγRIIb signaling is sensitive to different levels of sialylation. Specifically, a clear dose response is observed with S2-IVIg, with significant increase in FcγRIIb phosphorylation. There was a ~10-fold increase over the control at concentrations as low as 250 µg/mL, whereas IVIg required ~1000 µg/mL to show a ~10-fold increase over the control. Minimal FcγRIIb phosphorylation was observed for desialylated IVIg, even at the highest concentrations tested.

tAkt or β-Actin were used as loading and normalization controls. Supplementary Figure S1 demonstrates the suitability of either of these controls as they do not change with any treatments, and similar trends were observed when comparing phopho FcγRIIb signal with tAkt or β-actin. Since tAkt (~57 kDa) could be blotted on the same gel as phospho FcγRIIb (~41 kDa), we chose it for most blots for more consistent normalization of the protein load.

To our knowledge, this is the first report of FcγRIIb activation observed strictly under the conditions in which IVIg Fcs are sialylated and presented in a polymeric fashion.

### 3.3. TNFα/Anti-TNFα Immune Complexes Lead to FcγRIIb Signaling Only When Antibodies Are Sialylated

**Figure 3:**
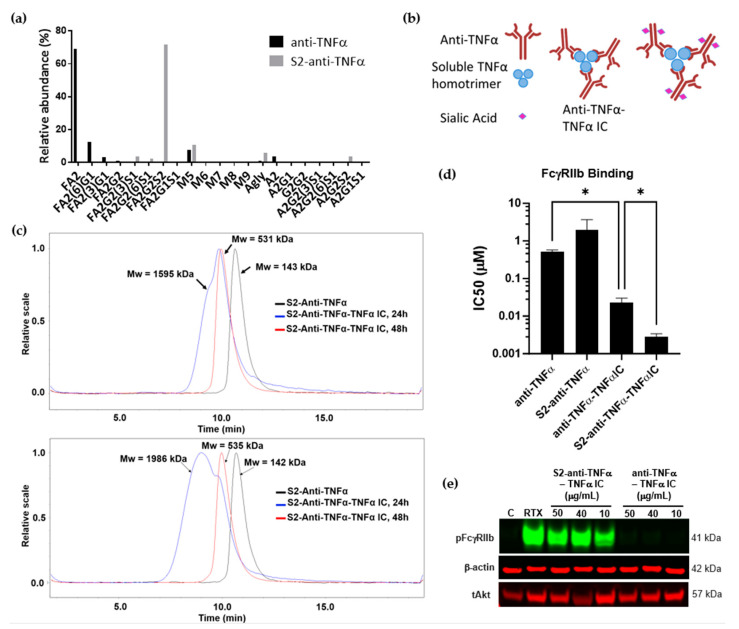
TNFα-(S2) anti TNFα IC formation, binding to and induction of FcyRIIb signaling. (**a**) N-glycopeptide analysis of anti-TNFα antibody and S2-anti-TNFα antibody was performed using LC-MS/MS. Oxford nomenclature for N-linked glycans: all N-glycans have two core GlcNAcs; F at the start of the abbreviation = core fucose; Ax, number x = the number of antenna (GlcNAc) on trimannosyl core; Gx, x = number of linked galactose on antenna; Sx, x = number of sialic acids linked to galactose; Mx = high mannose x. (**b**) Schematic of anti-TNFα-TNFα and S2-anti TNFα-TNFα ICs. (**c**) SEC-MALS estimation of average molecular weights of anti-TNFα and sialylated anti-TNFα and the corresponding ICs. (**d**) Relative binding of anti-TNFα/S2-anti-TNFα and ICs to FcγRIIb measured using cell-based homogeneous time-resolved fluorescence competition assays (TR-FRET CISBIO); error bars are standard error of the mean (SEM), *n* = 3. (**e**) Western blot of phospho FcγRIIb after 30 min stimulation of Daudi cells with rituximab at 5 µg/mL (positive control), anti-TNFα- TNFα immune complex (TNF IC) or sialylated anti-TNFα- TNFα IC (S2-TNF IC) at doses 50, 40, 10 µg/mL; representative data from *n* = 3 independent experiments. tAkt and β-actin were used as loading controls. Description of statistical methods can be found in the methods section. * *p* < 0.05.

The interplay between IgG-Fc sialylation and IgG valency has not been thoroughly explored in the past. To that end, an IC of TNFα/anti-TNFα was studied as the valency can be easily controlled and monitored. Fc sialylation was introduced into the recombinant anti-TNFα antibody (adalimumab) using an in vitro enzymatic method reported previously [13]. N-glycopeptide analysis was employed to examine the Fc glycosylation profile of anti-TNFα and a highly sialylated anti-TNFα (S2-anti-TNFα). The analysis confirmed full sialylation of complex-type, di-sialylated N-glycans in S2-anti-TNFα, which was absent in the non-sialylated anti-TNFα (Figure 3a).

Adalimumab antibody forms a trimeric complex when bound to TNFα, with three Fab anti-TNFα domains bound to a TNFα trimer [33] (schematic in Figure 3b). These interactions were simulated by incubating adalimumab with the TNFα antigen, and it was observed that a larger complex was formed within the first 24 h (~1595 kDa), which equilibrated by 48 h. The stable complex formed at 48 h was estimated to be ~531 kDa in molecular weight, roughly corresponding to the size of a trimeric complex (three TNFα molecules, each 17 kDa in size and three anti-TNFα antibodies, each 150 kDa; Figure 3c top panel). Similarly, sialylated anti-TNFα IC was successfully generated, showing that IgG-Fc sialylation did not negatively impact IC formation (Figure 3c bottom panel). It is well understood that valency can have a significant effect on the low affinity interactions between IgG-Fc and its low affinity FcγRs. These interactions are not only modulated by the valency of the Fc but also by the cellular density of the FcγRs. Therefore, it was necessary to evaluate the binding consequences of different glycoforms of monomeric anti-TNFα and trimeric TNFα/anti-TNFα ICs on the cells expressing low affinity FcγRIIb. As expected, trivalent IC exhibited a ~23-fold increase in binding to FcγRIIb receptor compared with the monovalent anti-TNFα molecule. Addition of sialylation to this trivalent molecule resulted in a significant (*p* = 0.031) ~8-fold increase in FcγRIIb binding ability compared with the non-sialylated IC (Figure 3d). We ensured that introducing sialylation to the monovalent anti-TNFα molecule did not obstruct its ability to bind all FcγRs (Supplementary Figure S2).

It is unclear from the current literature whether small differences in IgG binding avidity translate into functional activity of FcγRs. Despite the limited impact of sialylation in monovalent and trivalent forms on FcγRIIb binding, it was further explored whether there is an effect on FcγRIIb signaling. As shown in Figure 3e, Daudi B cells treated with TNFα-anti TNFα immune complex resulted in a dramatic increase in FcγRIIb phosphorylation only when the IC was sialylated (S2-TNF IC), whilst no phosphorylation of FcγRIIb was observed in the absence of the Fc sialylation (TNF IC). Interestingly, despite the observed significant increase in TNFα-anti TNFα IC binding to FcγRIIb compared with the monovalent anti-TNFα (Figure 3d), there was no FcγRIIb activation in the absence of sialic acid residues in the Fc chains. Similarly, monomeric sialylated anti-TNFα did not induce FcγRIIb signaling (Supplementary Figure S3). Therefore, although sialylation and valency alone have no impact on FcγRIIb signaling, when combined in the form of a sialylated immune complex, a dramatic increase in FcγRIIb phosphorylation can be achieved.

### 3.4. Increase in Fc Valency Significantly Impacts FcγRIIb Signaling Only in the Presence of Fc Sialylation

**Figure 4:**
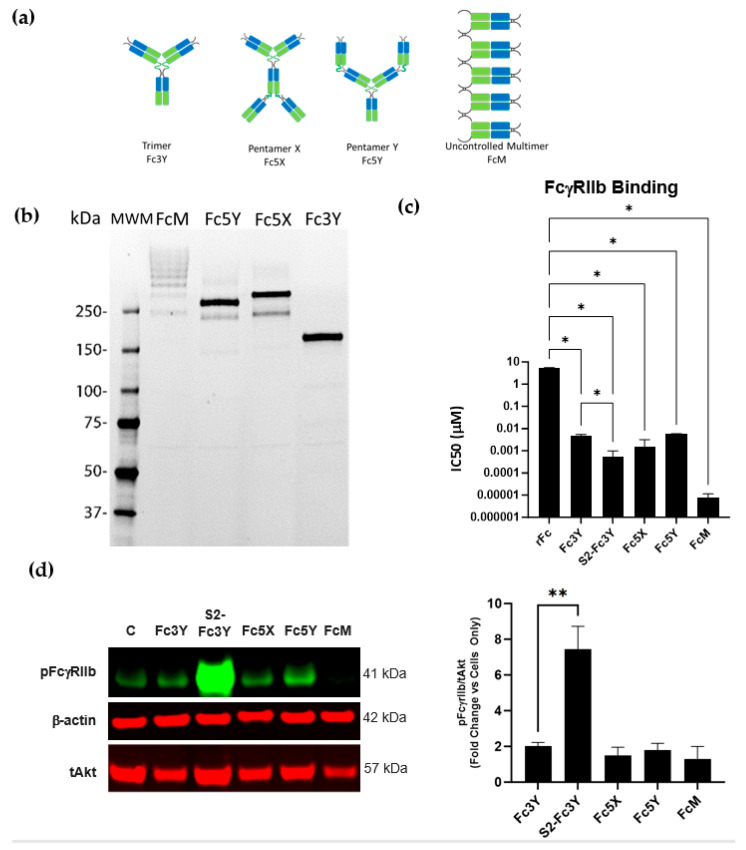
Multivalent Fc molecules do not induce FcγRIIb signaling unless they are sialylated. (**a**) Schematic representation of recombinant multivalent molecules. Blue regions correspond to the IgG1 CH3 domain. Green regions correspond to the IgG1 CH4 domain. (**b**) Multivalent molecules resolved on SDS-PAGE gel. (**c**) Multivalent Fc molecules binding to FcγRIIb measured by TR-FRET CISBIO. (**d**) Left: Western blot of phospho FcγRIIb after 30 min stimulation of Daudi cells with 100 µg/mL Fc3Y, Fc5X, Fc5Y, FcM and S2-Fc3Y; right: densitometry of the Western blot images across *n* = 5 independent experiments. tAkt and β-actin were used as loading controls. Description of statistical methods can be found in the methods section. * *p* < 0.05, ** *p* < 0.005.

To better understand the structure–activity relationship of Fc sialylation and IgG-Fc valency on FcγRIIb signaling, an antigen-independent system was employed by generating a panel of multivalent Fc molecules of discrete valency. Specifically, since it has been reported that FcγR signaling can be enhanced by increasing clustering of the cell surface receptors, it begs the question whether Fc molecules with higher valency could increase FcγRIIb signaling. Since sialylated anti-TNFα IC showed a small increase in binding to FcγRIIb in comparison with the non-sialylated IC, we thought it was important to evaluate if increasing binding to FcγRIIb is sufficient to explain the observed FcγRIIb signaling with the sialylated anti-TNFα IC. The panel of multivalent Fc molecules are denoted as Fc3Y (trimer), sialylated Fc3Y (S2-Fc3Y), Fc5X (pentamer X shape), Fc5Y (pentamer Y shape) and FcM (uncontrolled multimer), indicating the number of Fc domains contained in each (Figure 4a). Molecular weight was confirmed by resolving each oligomer on non-reducing sodium dodecyl sulfate polyacrylamide gel electrophoresis (SDS-PAGE) gel (Figure 4b). To address the effect on FcγRIIb binding, the avidity of recombinantly generated reagents was measured. As expected, an increase in Fc valency in all multivalent Fc constructs led to a significant increase in binding to FcγRIIb, with Fc3Y showing ~1000-fold greater binding affinity compared with the monovalent recombinant Fc. Pentamer Fc5X showed ~3500-fold higher binding avidity, while Y shaped pentamer bound similarly to Fc3Y, exhibiting ~950-fold binding compared with recombinant Fc (rFc). However, uncontrolled multimer, which contains many Fc chains, showed a dramatic increase in binding, amounting to ~600,000-fold higher than that of rFc (Figure 4c). Similar to our results with the FcγRIIb binding of the Anti-TNFα- TNFα IC, S2-Fc3Y had a significant (*p* = 0.046) ~9-fold difference in FcγRIIb binding compared with the non-sialylated Fc3Y. To our surprise, neither of the multivalent molecules tested were able to induce significant FcγRIIb signaling despite such dramatic increases in FcγRIIb binding avidity (Figure 4d). Strikingly, sialylated trimer (S2-Fc3Y) induced a dramatic increase in FcγRIIb signaling. These results are consistent with the Fc sialylation-dependent FcγRIIb signaling observed for polymerically presented sialylated IVIg and anti-TNFα IC.

**Figure 5:**
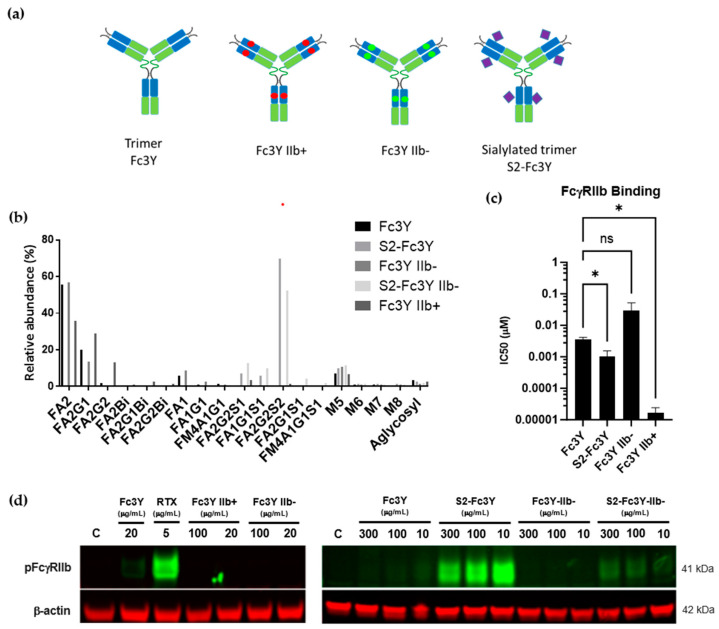
Sialylation and FcγRIIb binding in multivalent Fc molecules are critical to induce FcγRIIb signaling. (**a**) Schematic of multivalent molecules. Blue regions correspond to the IgG1 CH3 domain. Green regions correspond to the IgG1 CH4 domain. Red dots indicate the FcγRIIb affinity enhancing mutations. Green dots indicate FcγRIIb affinity decreasing mutations. Purple diamonds indicate Fc Sialylation (FA2G2S2) at Asn297. (**b**) N-glycopeptide profile of the multivalent Fc molecules was obtained using LC-MS/MS. Oxford nomenclature for N-linked glycans: all N-glycans have two core GlcNAcs; F at the start of the abbreviation = core fucose; Ax, number x = the number of antenna (GlcNAc) on trimannosyl core; Gx, x = number of linked galactose on antenna; Sx, x = number of sialic acids linked to galactose; Mx = high mannose x. (**c**) Multivalent Fc molecules binding to FcγRIIb measured by TR-FRET CISBIO. *n* = 3 independent experiments. (**d**) Western blot of phospho FcγRIIb after 30 min stimulation of Daudi cells with each multivalent Fc molecule; representative data from *n* = 3 independent observations. β-actin was used as loading control. Description of statistical methods can be found in the methods section. * *p* < 0.05, ns = non-significant.

To further expand on the studies above, a range of trivalent IgG-Fc molecules with enhanced (Fc3Y IIb+) or decreased (Fc3Y IIb-) binding to FcγRIIb as well as sialylated Fc3Y (S2-Fc3Y) and sialylated Fc3Y with decreased binding to FcγRIIb (S2-Fc3Y IIb-) were generated. (A schematic of the constructs is shown in the Figure 5a). N-glycopeptide profile analysis confirmed effective enzymatic sialylation of the S2-Fc3Y and S2-Fc3Y IIb- constructs (Figure 5b). TR-FRET Cisbio data confirmed FcγRIIb binding of Fc3Y IIb+ and S2-Fc3Y constructs, whilst Fc3Y IIb-mutant exhibited reduced ability to bind FcγRIIb (Figure 5c). Surprisingly, even with the increased binding to FcγRIIb, Fc3Y IIb+ did not result in enhanced FcγRIIb signaling (Figure 5d, left panel). By stark contrast, sialylated Fc3Y (S2-Fc3Y) induced potent FcγRIIb phosphorylation over a range of doses tested (Figure 5d, right panel and Figure 4d). Furthermore, direct binding to FcγRIIb was also necessary for this effect to occur as sialylated Fc3Y molecules engineered to lose FcγRIIb binding ability did not result in FcγRIIb phosphorylation (Figure 5d, S2-Fc3Y IIb- lane). Therefore, both Fc sialylation of ICs and intact binding to FcγRIIb are essential for successful FcγRIIb phosphorylation.

### 3.5. FcgRIIb Singaling in PBMCs Is Dependent on Fc Valency and Sialylation

**Figure 6:**
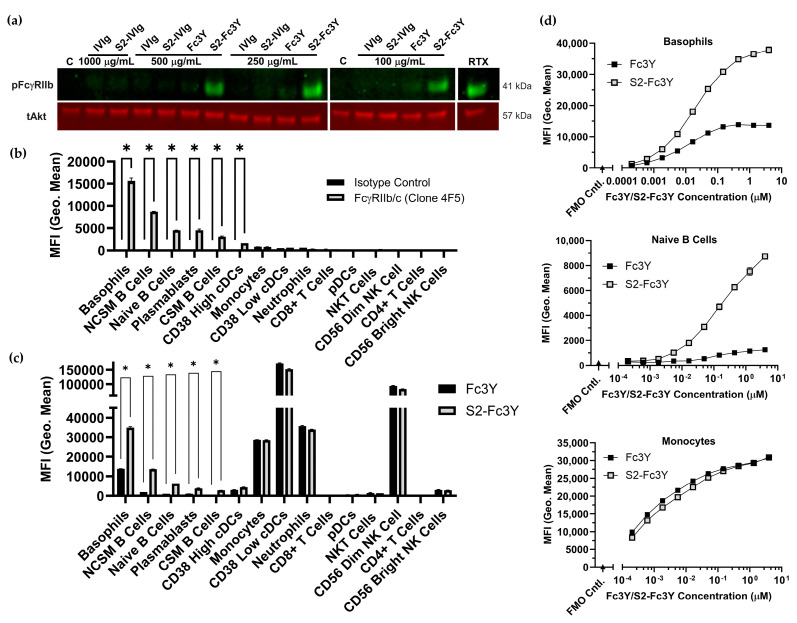
Fc valency and sialylation required for FcγRIIb activation and increased binding in PBMCs. (**a**) Western blot of phospho FcγRIIb after 30 min stimulation of human PBMCs with monomeric IVIg and multivalent Fc molecules; representative data from *n* = 3 independent donors. tAkt was used as loading control. (**b**) FcγRIIb expression in human PBMCs measured by flow cytometry using anti-FcγRIIb/c antibody clone 4F5. Data are representative of *n* = 3 independent donors. (**c**) Binding of VivoTag 645-labeled Fc3Y and S2-Fc3Y (66 μg/mL) to human PBMCs measured by flow cytometry. Data are representative of *n* = 3 independent donors. (**d**) Binding of dose responses of VivoTag 645-labeled Fc3Y and S2-Fc3Y to human PBMCs measured by flow cytometry. Data are representative of *n* = 3 independent donors. NCSM B Cells = non-class switched memory B cells, CSM B Cells = class switched memory B cells, cDCs = conventional dendritic cells, pDCs = plasmacytoid dendritic cells. Description of statistical methods can be found in the methods section. * *p* < 0.05.

To determine if valency and Fc sialylation-dependent FcγRIIb phosphorylation occurs in a more physiologic setting, we probed the activity of multi-valent Fc constructs using human peripheral blood mononuclear cells (PBMCs). While monomeric IVIg, S2-IVIg, and Fc3Y had no effect, S2-Fc3Y induced FcγRIIb phosphorylation over a range of doses in PBMCs (Figure 2a). Multivalent plate-bound IVIg and S2-IVIg were also found to induce FcγRIIb phosphorylation in PBMCs (Supplementary Figure S4). These results in PBMCs further confirmed our findings in Daudi cells that both Fc valency and sialylation are required for the induction of FcγRIIb signaling. To better understand which cell types may be contributing to the observed phospho FcγRIIb signal, we measured FcγRIIb expression via flow cytometry on PBMCs using an FcγRIIb/c-specific antibody as part of a 14-color panel to identify major cell subsets. The gating strategy and antibody panel information can be found in Supplementary Figure S5. Results from a single donor are shown in Figure 6b, with results for an additional two donors shown in Supplementary Figure S6. As expected, nearly all the B cell subsets expressed FcγRIIb, albeit to varying levels, with non-class switched IgD+ CD27+ memory B cells (NCSM B Cells) expressing the highest levels. An HLA-DR+, CD11c+, CD38++ dendritic cell subset (CD38 High cDCs) displayed low but significant expression of FcγRIIb. Basophils displayed the highest level of FcγRIIb expression.

We next determined which cell types the Fc3Y and S2-Fc3Y constructs interacted with in PBMCs. Both constructs were labeled with VivoTag 645 and were used to stain PBMCs for flow cytometry analysis. The degree of dye labeling was found to be comparable between the two constructs. Compared with the non-sialylated Fc3Y, S2-Fc3Y was found to interact more strongly with cells expressing FcγRIIb such as basophils and B cells (Figure 6c). Fc3Y and S2-Fc3Y had equivalent binding to other cells such as monocytes, DCs, neutrophils and NK cells. These cells are all known to express other Fc gamma receptors but no FcγRIIb based on the results in Figure 6b, suggesting that binding to these cells is dominated by activating Fc gamma receptors. Figure 6d demonstrates that S2-Fc3Y’s increased binding to FcγRIIb+ cells across a wide range of doses relative to Fc3Y, with equivalent binding to FcγRIIb- cells such as monocytes. Dose responses for each cell subset can be found in Supplementary Figure S7. Together, these data demonstrate that Fc sialylation and multi-valency are required for FcγRIIb signaling in primary human cells. In addition, Fc sialylation appears to permit increased binding to FcγRIIb-expressing cells compared with non-sialylated constructs.

## 4. Discussion

It is generally accepted that the pro- and anti-inflammatory activity of IgGs can be modulated by different properties of IgG-Fc, such as subclass, glycosylation and valency, as reviewed in detail previously [34,35]. IgG subclass can direct the interaction profile of IgG-Fc with the different FcγRs, and IgG glycosylation may impact the interaction with type I and type II Fc-receptors [36]. An increase in IgG valency results in higher avidity for FcγRs and subsequent clustering of these receptors, which in certain cases, can lead to downstream signaling [37]. Characterizing the structure–activity relationship of antibodies in vitro often suffers from limitations such as presentation of the antibody in a different format from its natural in vivo orientation, especially after coordination with cognate antigens. In fact, Nimmerjahn and colleagues studied the effects of IgG Fc glycosylation using an antibody containing only a monosaccharide residue in the Fc. Their studies showed that the activity of minimally glycosylated antibodies is not predicted by in vitro assays based on a monomeric antibody–FcγR interaction analysis; in vitro assay systems using immune complexes proved to be more suitable for predicting IgG activity in vivo [29].

FcγRIIb has been shown to suppress the pro-inflammatory signaling mediated by activating FcγRs through its ITIM domain, and in that way, it determines the threshold of cellular activation [38]. In B cells, the antigen serves as a scaffold for the formation of immune complexes that can activate the B cell receptor (BCR). B cell FcγRIIb modulates signaling of the BCR by co-ligation of FcγRIIb and the BCR, inhibiting pro-inflammatory signals in a SHIP-mediated manner, resulting in reduced B-cell activation and proliferation [39,40,41,42]. FcγRIIb homo-ligation, on the other hand, has been shown to induce B cell apoptosis [43,44]. Activation of the inhibitory FcγRIIb has been demonstrated with anti-FcγRIIb antibodies which can amplify the inhibitory signaling of FcγRIIb after co-ligation of BCR and FcγRIIb by immune complexes [45]. Therefore, modulation of FcγRIIb inhibitory function is an attractive approach for the development of an innovative immunomodulatory drug.

It has been previously shown that Fc glycosylation can impact the modulation of Fc-receptors. IgG-Fc sialylation has been shown to drive the anti-inflammatory activity of IVIg through modulation of FcγRIIb in vivo [15]. In this work, we demonstrated that Fc sialylation has the potential to induce FcγRIIb signaling; however, this effect cannot be observed in vitro unless the IgGs are displayed in a multimeric format; i.e., simulating the arrangement of the antibodies in an immune complex. Our work presented here points to the unique way FcγRIIb activation in B cells is achieved through the interplay between valency and sialylation. Our data demonstrate, for the first time, the activation of FcγRIIb by sialylated Fcs in IgGs only when presented in polymeric formats. This was observed across two autoimmune disease therapeutics and engineered multivalent Fc molecules. Immune complexes of sialylated adalimumab and TNFα lead to a dramatic increase in FcγRIIb phosphorylation, while virtually no FcγRIIb phosphorylation was achieved with non-sialylated ICs. It was intriguing that the observation was binary, implying that sialylation is necessary for any FcγRIIb phosphorylation to occur, even in the presence of multiple Fcs that presumably aggregate FcγRIIb receptors together.

The extent to which sialylation is required was shown through our work using sialylated IVIg manipulated to form multivalent complexes by opsonizing bioparticles and adhering them to the culture plates (Figure 1 and Figure 2 respectively). The anti-inflammatory activity of IVIg that is FcγRIIb-dependent is not novel, and it has been shown to be associated with the fraction of sialylated IVIg in the IVIg preparation, among other mechanisms [4,11,14,16]. A few groups have proposed that IVIg induces anti-inflammatory activities by altering the relative surface expression of activating and inhibiting FcγRs in favor of the inhibitory FcγRIIb, thereby increasing the signal threshold necessary to activate immune cells [4,15]. However, the impact sialylated IVIg has on direct activation of FcγRIIb on immune cells was not demonstrated in any of these studies, perhaps due to the technical limitations. The data supporting the mechanistic impact of sialylated IVIg on FcγRIIb modulation stem from animal models, in which IVIg protected mice from pathogenic IgG, and this was dependent on FcγRIIb [11,18,19,46,47]. Furthermore, results of several studies showed that FcγRIIb is upregulated on innate immune effector cells and B cells after IVIg treatment in mice [11,17,19], and this was confirmed in patients with CIDP, in whom FcγRIIb expression levels on B cells were impaired. When examining the sialylation levels naturally present in IVIg pools, we demonstrated that ~20% of IVIg was sialylated, which in turn led to FcγRIIb phosphorylation when presented as a multivalent complex (Figure 1). However, the magnitude of pFcγRIIb induced by partially sialylated IVIg was significantly less potent compared with the tetra-sialylated IVIg at all doses tested, implying that FcγRIIb signaling is sensitive to the levels of sialylated Fcs present in the complex (Figure 2b). By contrast, when IVIg was enzymatically altered to be devoid of any sialic acid, FcγRIIb activation was impaired.

The requirement for Fc sialylation was further demonstrated by using engineered Fc molecules. The increase in Fc valency alone was not enough to induce FcγRIIb phosphorylation even if FcγRIIb binding affinity was ~100,000-fold higher compared with the monovalent Fc (Figure 4c FcM vs. rFc). Site-specific sialic acid in the Fc needs to be present in polyvalent complexes for FcγRIIb phosphorylation to occur. Conversely, sialylated multivalent complexes lose the ability to activate FcγRIIb if they are unable to bind to it, as shown by using S2-Fc3Y IIb- engineered to lose affinity for FcγRIIb (Figure 5). Finally, the FcγRIIb phosphorylation results obtained from Daudi cells were reconfirmed in primary human PBMCs using both S2-Fc3Y (Figure 6a) and plate-bound IVIg (Supplementary Figure S4). In addition, S2-Fc3Y exhibited increased binding to FcγRIIb-expressing cells but was comparable to Fc3Y in non-FcγRIIb-expressing cells (Figure 6b–d). These results add to the possibility of Fc sialylation and valency playing a role in FcγRIIb signaling in non-B cells.

Taken together, our data imply that clustering of FcγRIIb is a prerequisite to its activation but not sufficient in the absence of sialylation. In some way, sialic acid present on the Fcs of the polyvalent molecules enables FcγRIIb activation, potentially through co-ligation with other yet unidentified receptors. One possibility might be that FcγRIIb is co-aggregated with one of the sialic acid-binding, immunoglobulin-type lectin receptors (SIGLECs) or type II FcγRs. Indeed, it has been shown, albeit in an artificial system, that simultaneous engagement of FcγRIIb and CD22 SIGLEC leads to FcγRIIb phosphorylation and subsequently silenced B cells [48]. More recently, Seeling et al. identified a novel role for the C-type lectin Dectin-1 (CLEC7A) in IVIg’s ability to ameliorate bone erosion in mouse models of arthritis. Their findings suggest that Dectin-1 on monocytes modulates both FcγRIIb binding and ITIM phosphorylation via promotion of FcγRIIb membrane clustering [49]. Such findings highlight the numerous possible mechanisms by which IgGs can exert their anti-inflammatory activity.

## 5. Conclusions

Taken together, our work demonstrates the importance of the interplay between Fc sialylation and valency in IgG immune complexes. The physiological role of the FcγRIIb receptor as one of the main inhibitory receptors on B cells responsible for limiting B cell activation by immune complexes makes it a suitable target to modulate the pathogenic processes underlying many autoimmune diseases. The discovery of how important sialylation and valency are in activation of FcγRIIb can be leveraged when designing novel therapeutics to treat autoimmune diseases.

## Data Availability

All data generated or analyzed during this study are included in this published article.

## Supplementary Materials

The following supporting information can be downloaded at: https://www.mdpi.com/article/10.3390/cells12172130/s1. Figure S1: S2-IVIg induces FcγRIIb signaling in Daudi cells when presented in polymeric fashion. Figure S2: Relative binding of anti-TNFα/S2-anti-TNFα to FcγRs measured using cell-based homogeneous TR-FRET CISBIO. Figure S3: TNFα-anti TNFα IC when sialylated induced FcγRIIb signaling. Figure S4: S2-IVIg induces FcγRIIb signaling in PBMCs when presented in polymeric fashion. Figure S5: Gating strategy for PBMC flow cytometry experiments. Figure S6: FcγRIIb expression in human PBMCs from healthy donors. Figure S7: Dose response staining of human PBMCs with VivoTag 645-labeled Fc3Y and S2-Fc3Y. Table S1: Antibody panel used for PBMC flow cytometry experiments. Table S2: Gating strategy and cell type markers for PBMC flow cytometry experiments.

## References

[1] Hogarth P.M., Pietersz G.A. (2012). Fc receptor-targeted therapies for the treatment of inflammation, cancer and beyond. Nat. Rev. Drug Discov..

[2] Nimmerjahn F., Ravetch J.V. (2011). FcgammaRs in health and disease. Curr. Top. Microbiol. Immunol..

[3] Arnold J.N., Wormald M.R., Sim R.B., Rudd P.M., Dwek R.A. (2007). The impact of glycosylation on the biological function and structure of human immunoglobulins. Annu. Rev. Immunol..

[4] Schwab I., Nimmerjahn F. (2013). Intravenous immunoglobulin therapy: How does IgG modulate the immune system?. Nat. Rev. Immunol..

[5] Mimura Y., Church S., Ghirlando R., Ashton P.R., Dong S., Goodall M., Lund J., Jefferis R. (2000). The influence of glycosylation on the thermal stability and effector function expression of human IgG1-Fc: Properties of a series of truncated glycoforms. Mol. Immunol..

[6] Mimura Y., Sondermann P., Ghirlando R., Lund J., Young S.P., Goodall M., Jefferis R. (2001). Role of oligosaccharide residues of IgG1-Fc in Fc gamma RIIb binding. J. Biol. Chem..

[7] Jefferis R., Lund J. (2002). Interaction sites on human IgG-Fc for FcgammaR: Current models. Immunol. Lett..

[8] Sondermann P., Kaiser J., Jacob U. (2001). Molecular basis for immune complex recognition: A comparison of Fc-receptor structures. J. Mol. Biol..

[9] Nimmerjahn F., Ravetch J.V. (2006). Fcgamma receptors: Old friends and new family members. Immunity.

[10] Scallon B.J., Tam S.H., McCarthy S.G., Cai A.N., Raju T.S. (2007). Higher levels of sialylated Fc glycans in immunoglobulin G molecules can adversely impact functionality. Mol. Immunol..

[11] Kaneko Y., Nimmerjahn F., Ravetch J.V. (2006). Anti-inflammatory activity of immunoglobulin G resulting from Fc sialylation. Science.

[12] Schwab I., Mihai S., Seeling M., Kasperkiewicz M., Ludwig R.J., Nimmerjahn F. (2014). Broad requirement for terminal sialic acid residues and FcgammaRIIB for the preventive and therapeutic activity of intravenous immunoglobulins in vivo. Eur. J. Immunol..

[13] Washburn N., Schwab I., Ortiz D., Bhatnagar N., Lansing J.C., Medeiros A., Tyler S., Mekala D., Cochran E., Sarvaiya H. (2015). Controlled tetra-Fc sialylation of IVIg results in a drug candidate with consistent enhanced anti-inflammatory activity. Proc. Natl. Acad. Sci. USA.

[14] Tackenberg B., Jelcic I., Baerenwaldt A., Oertel W.H., Sommer N., Nimmerjahn F., Lunemann J.D. (2009). Impaired inhibitory Fcgamma receptor IIB expression on B cells in chronic inflammatory demyelinating polyneuropathy. Proc. Natl. Acad. Sci. USA.

[15] Anthony R.M., Kobayashi T., Wermeling F., Ravetch J.V. (2011). Intravenous gammaglobulin suppresses inflammation through a novel T(H)2 pathway. Nature.

[16] Anthony R.M., Nimmerjahn F., Ashline D.J., Reinhold V.N., Paulson J.C., Ravetch J.V. (2008). Recapitulation of IVIG anti-inflammatory activity with a recombinant IgG Fc. Science.

[17] Bruhns P., Samuelsson A., Pollard J.W., Ravetch J.V. (2003). Colony-stimulating factor-1-dependent macrophages are responsible for IVIG protection in antibody-induced autoimmune disease. Immunity.

[18] Huang H.S., Sun D.S., Lien T.S., Chang H.H. (2010). Dendritic cells modulate platelet activity in IVIg-mediated amelioration of ITP in mice. Blood.

[19] Samuelsson A., Towers T.L., Ravetch J.V. (2001). Anti-inflammatory activity of IVIG mediated through the inhibitory Fc receptor. Science.

[20] Hurez V., Kaveri S.V., Mouhoub A., Dietrich G., Mani J.C., Klatzmann D., Kazatchkine M.D. (1994). Anti-CD4 activity of normal human immunoglobulin G for therapeutic use. (Intravenous immunoglobulin, IVIg). Ther. Immunol..

[21] Lamoureux J., Aubin E., Lemieux R. (2003). Autoimmune complexes in human serum in presence of therapeutic amounts of intravenous immunoglobulins. Blood.

[22] Lamoureux J., Aubin E., Lemieux R. (2004). Autoantibodies purified from therapeutic preparations of intravenous immunoglobulins (IVIg) induce the formation of autoimmune complexes in normal human serum: A role in the in vivo mechanisms of action of IVIg?. Int. Immunol..

[23] Svenson M., Hansen M.B., Ross C., Diamant M., Rieneck K., Nielsen H., Bendtzen K. (1998). Antibody to granulocyte-macrophage colony-stimulating factor is a dominant anti-cytokine activity in human IgG preparations. Blood.

[24] Jerzak M., Rechberger T., Gorski A. (2000). Intravenous immunoglobulin therapy influences T cell adhesion to extracellular matrix in women with a history of recurrent spontaneous abortions. Am. J. Reprod. Immunol..

[25] Rossi F., Kazatchkine M.D. (1989). Antiidiotypes against autoantibodies in pooled normal human polyspecific Ig. J. Immunol..

[26] Takei S., Arora Y.K., Walker S.M. (1993). Intravenous immunoglobulin contains specific antibodies inhibitory to activation of T cells by staphylococcal toxin superantigens [see comment]. J. Clin. Investig..

[27] Teeling J.L., Jansen-Hendriks T., Kuijpers T.W., de Haas M., van de Winkel J.G., Hack C.E., Bleeker W.K. (2001). Therapeutic efficacy of intravenous immunoglobulin preparations depends on the immunoglobulin G dimers: Studies in experimental immune thrombocytopenia. Blood.

[28] Zhuang Q., Mazer B. (2001). Inhibition of IgE production in vitro by intact and fragmented intravenous immunoglobulin. J. Allergy Clin. Immunol..

[29] Kao D., Danzer H., Collin M., Gross A., Eichler J., Stambuk J., Lauc G., Lux A., Nimmerjahn F. (2015). A Monosaccharide Residue Is Sufficient to Maintain Mouse and Human IgG Subclass Activity and Directs IgG Effector Functions to Cellular Fc Receptors. Cell Rep..

[30] Lux A., Yu X., Scanlan C.N., Nimmerjahn F. (2013). Impact of immune complex size and glycosylation on IgG binding to human FcgammaRs. J. Immunol..

[31] Lim S.H., Vaughan A.T., Ashton-Key M., Williams E.L., Dixon S.V., Chan H.T., Beers S.A., French R.R., Cox K.L., Davies A.J. (2011). Fc gamma receptor IIb on target B cells promotes rituximab internalization and reduces clinical efficacy. Blood.

[32] Siragam V., Brinc D., Crow A.R., Song S., Freedman J., Lazarus A.H. (2005). Can antibodies with specificity for soluble antigens mimic the therapeutic effects of intravenous IgG in the treatment of autoimmune disease?. J. Clin. Investig..

[33] Santora L.C., Kaymakcalan Z., Sakorafas P., Krull I.S., Grant K. (2001). Characterization of noncovalent complexes of recombinant human monoclonal antibody and antigen using cation exchange, size exclusion chromatography, and BIAcore. Anal. Biochem..

[34] Beneduce C., Kurtagic E., Bosques C.J. (2019). Anti-inflammatory Activity of IgG-Fc. Curr. Top. Microbiol. Immunol..

[35] Nimmerjahn F., Vidarsson G., Cragg M.S. (2023). Effect of posttranslational modifications and subclass on IgG activity: From immunity to immunotherapy. Nat. Immunol..

[36] Pincetic A., Bournazos S., DiLillo D.J., Maamary J., Wang T.T., Dahan R., Fiebiger B.M., Ravetch J.V. (2014). Type I and type II Fc receptors regulate innate and adaptive immunity. Nat. Immunol..

[37] Maenaka K., van der Merwe P.A., Stuart D.I., Jones E.Y., Sondermann P. (2001). The human low affinity Fcgamma receptors IIa, IIb, and III bind IgG with fast kinetics and distinct thermodynamic properties. J. Biol. Chem..

[38] Boruchov A.M., Heller G., Veri M.C., Bonvini E., Ravetch J.V., Young J.W. (2005). Activating and inhibitory IgG Fc receptors on human DCs mediate opposing functions. J. Clin. Investig..

[39] Chu S.Y., Vostiar I., Karki S., Moore G.L., Lazar G.A., Pong E., Joyce P.F., Szymkowski D.E., Desjarlais J.R. (2008). Inhibition of B cell receptor-mediated activation of primary human B cells by coengagement of CD19 and FcgammaRIIb with Fc-engineered antibodies. Mol. Immunol..

[40] Heyman B. (2003). Feedback regulation by IgG antibodies. Immunol. Lett..

[41] Kiener P.A., Lioubin M.N., Rohrschneider L.R., Ledbetter J.A., Nadler S.G., Diegel M.L. (1997). Co-ligation of the antigen and Fc receptors gives rise to the selective modulation of intracellular signaling in B cells. Regulation of the association of phosphatidylinositol 3-kinase and inositol 5′-phosphatase with the antigen receptor complex. J. Biol. Chem..

[42] Ono M., Okada H., Bolland S., Yanagi S., Kurosaki T., Ravetch J.V. (1997). Deletion of SHIP or SHP-1 reveals two distinct pathways for inhibitory signaling. Cell.

[43] Xiang Z., Cutler A.J., Brownlie R.J., Fairfax K., Lawlor K.E., Severinson E., Walker E.U., Manz R.A., Tarlinton D.M., Smith K.G. (2007). FcgammaRIIb controls bone marrow plasma cell persistence and apoptosis. Nat. Immunol..

[44] Pearse R.N., Kawabe T., Bolland S., Guinamard R., Kurosaki T., Ravetch J.V. (1999). SHIP recruitment attenuates Fc gamma RIIB-induced B cell apoptosis. Immunity.

[45] Rieth N., Carle A., Muller M.A., ter Meer D., Direnberger C., Pohl T., Sondermann P. (2014). Characterization of SM201, an anti-hFcgammaRIIB antibody not interfering with ligand binding that mediates immune complex dependent inhibition of B cells. Immunol. Lett..

[46] Akilesh S., Petkova S., Sproule T.J., Shaffer D.J., Christianson G.J., Roopenian D. (2004). The MHC class I-like Fc receptor promotes humorally mediated autoimmune disease. J. Clin. Investig..

[47] Crow A.R., Song S., Freedman J., Helgason C.D., Humphries R.K., Siminovitch K.A., Lazarus A.H. (2003). IVIg-mediated amelioration of murine ITP via FcgammaRIIB is independent of SHIP1, SHP-1, and Btk activity. Blood.

[48] Mihaylova N., Voynova E., Tchorbanov A., Dolashka-Angelova P., Bayry J., Devreese B., Kaveri S., Vassilev T. (2009). Simultaneous engagement of FcgammaIIb and CD22 inhibitory receptors silences targeted B cells and suppresses autoimmune disease activity. Mol. Immunol..

[49] Seeling M., Pohnl M., Kara S., Horstmann N., Riemer C., Wohner M., Liang C., Bruckner C., Eiring P., Werner A. (2023). Immunoglobulin G-dependent inhibition of inflammatory bone remodeling requires pattern recognition receptor Dectin-1. Immunity.

