# Supplementary material for: Inhibitory Fc-Gamma IIb Receptor Signaling Induced by Multivalent IgG-Fc Is Dependent on Sialylation
Source: Cells. 2023 Aug 23;12(17):2130. doi: 10.3390/cells12172130 (PMC10486564; doi:10.3390/cells12172130)
Supplement: Supplementary file 1 [file cells-12-02130-s001.zip › cells-2527703-supplementary.pdf]

# Inhibitory Fc-gamma IIb Receptor Signaling Induced by Multivalent IgG-Fc is Dependent on Sialylation

**Christopher Beneduce[1,2], Stephanie Nguyen[1], Nathaniel Washburn[1,2], John Schaeck[1], Robin Meccariello[1,2], Kimberly Holte[1], Daniel Ortiz[1], Anthony M. Manning[1], Carlos J. Bosques[1] and Elma Kurtagic[1,2]***

[1] Momenta Pharmaceuticals Inc, Cambridge, MA, USA
[2] Janssen Pharmaceutical Companies of Johnson & Johnson, Cambridge, MA, USA
* Correspondence: ekurtagi@its.jnj.com.com

## Supplemental Figures:

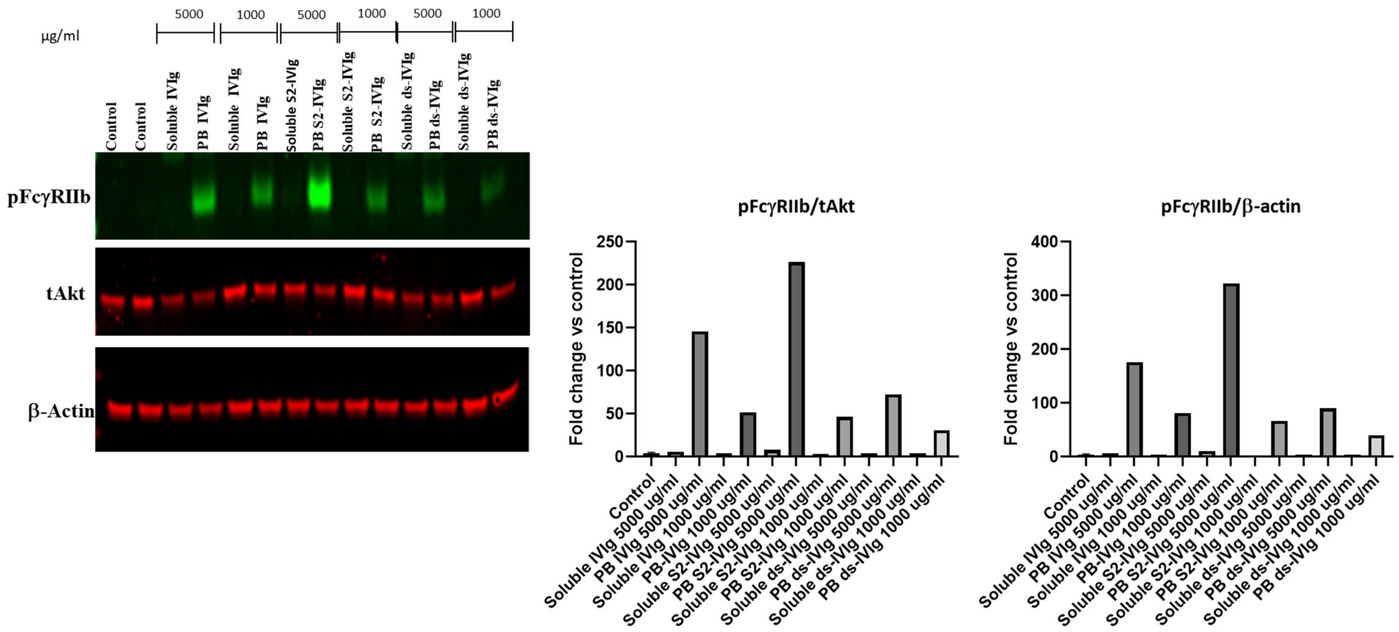


**Supplementary Figure S1. S2-IVIg induces FcγRIIb signaling in Daudi cells when presented in polymeric fashion.** Left: Western blot of phospho FcγRIIb, tAkt and β-actin after 30 min stimulation of Daudi cells with soluble or plate-bound IVIg, S2-IVIg or ds-IVIg. Right panel: densitometry of the western blots showing pFcγRIIb signal over tAkt or β-actin, resulting in the same relative fold changes.

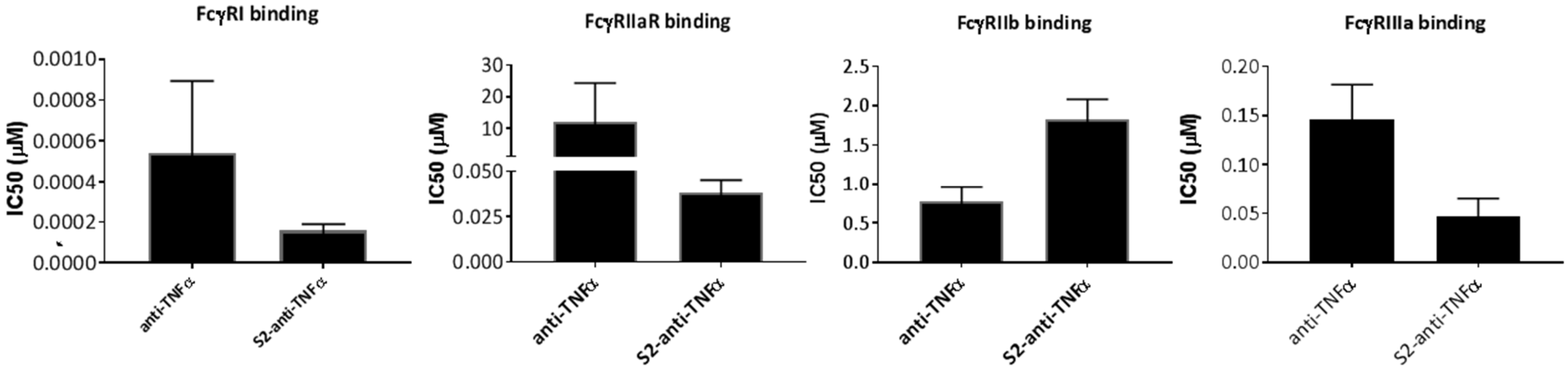


**Supplementary Figure S2. R**elative binding of anti-TNF$\alpha$/S2-anti-TNF$\alpha$ to FcγRs measured using cell-based homogeneous TR-FRET CISBIO.

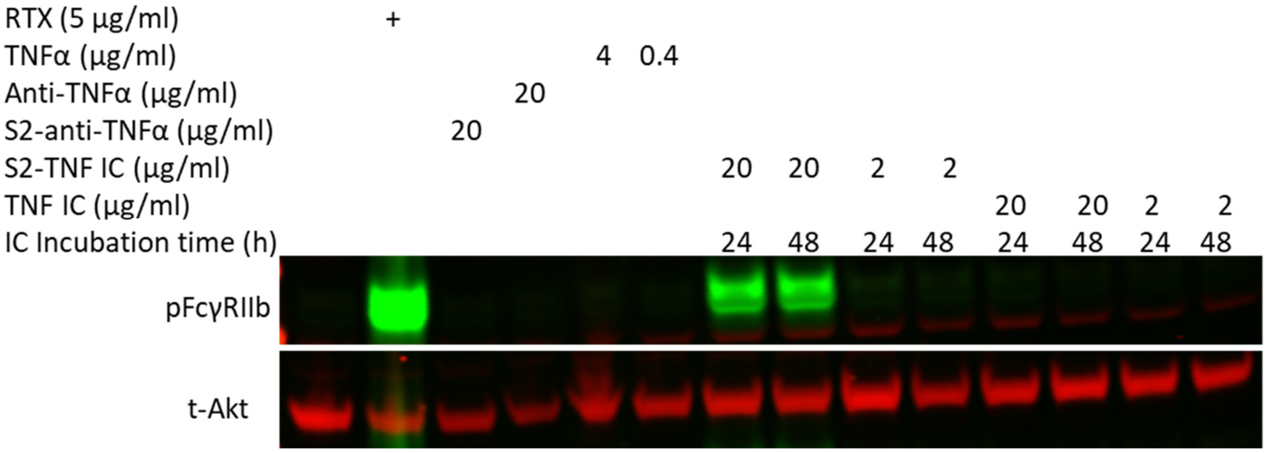


**Supplementary Figure S3. TNF$\alpha$-anti TNF$\alpha$ IC when sialylated, induced Fc$\gamma$RIIb signaling.** Western blot of phospho Fc$\gamma$RIIb and β-actin after 30 min stimulation of Daudi cells with rituximab (5 μg/ml), TNF$\alpha$ (4 and 0.4 μg/ml), 20 μg/ml anti-TNF$\alpha$ and S2-anti-TNF$\alpha$, TNF$\alpha$-anti TNF$\alpha$ immune complex (TNF IC) or sialylated anti-TNF$\alpha$-TNF$\alpha$ IC (S2-TNF IC) formed over 24 or 48 hr incubation time, at doses 20 and 2 μg/ml.

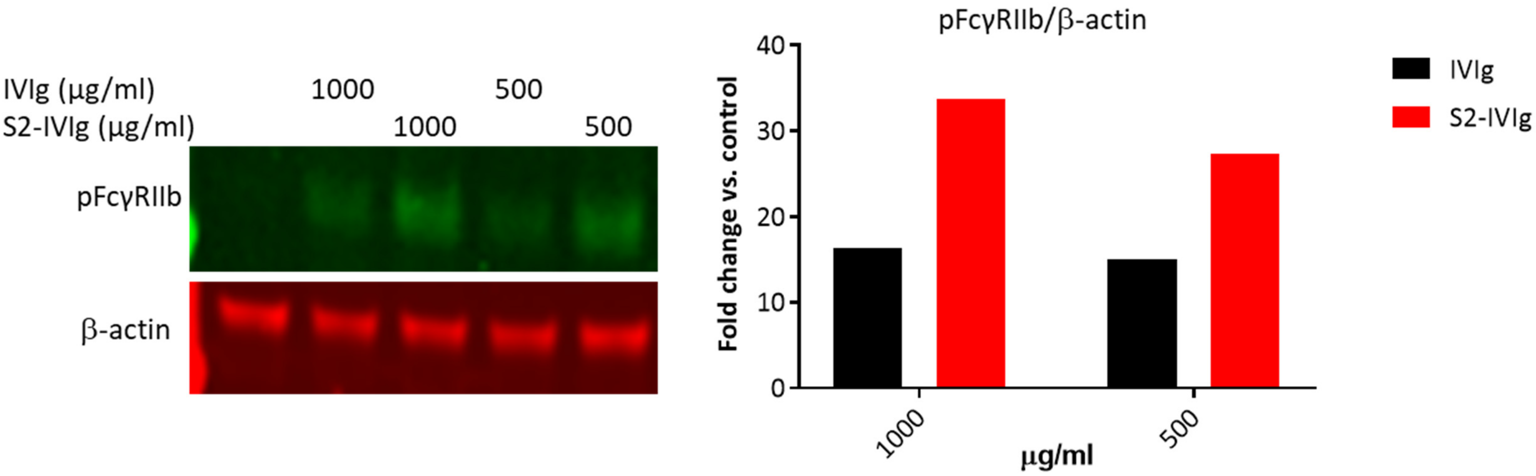


**Supplementary Figure S4: S2-IVIg induces Fc$\gamma$RIIb signaling in PBMCs when presented in polymeric fashion.** Left: Western blot of phospho Fc$\gamma$RIIb and β-actin after 30 min stimulation of freshly isolated PBMC cells with plate-bound IVIg or S2-IVIg (1000, 500 μg/ml). Right: densitometry of Western blot image.

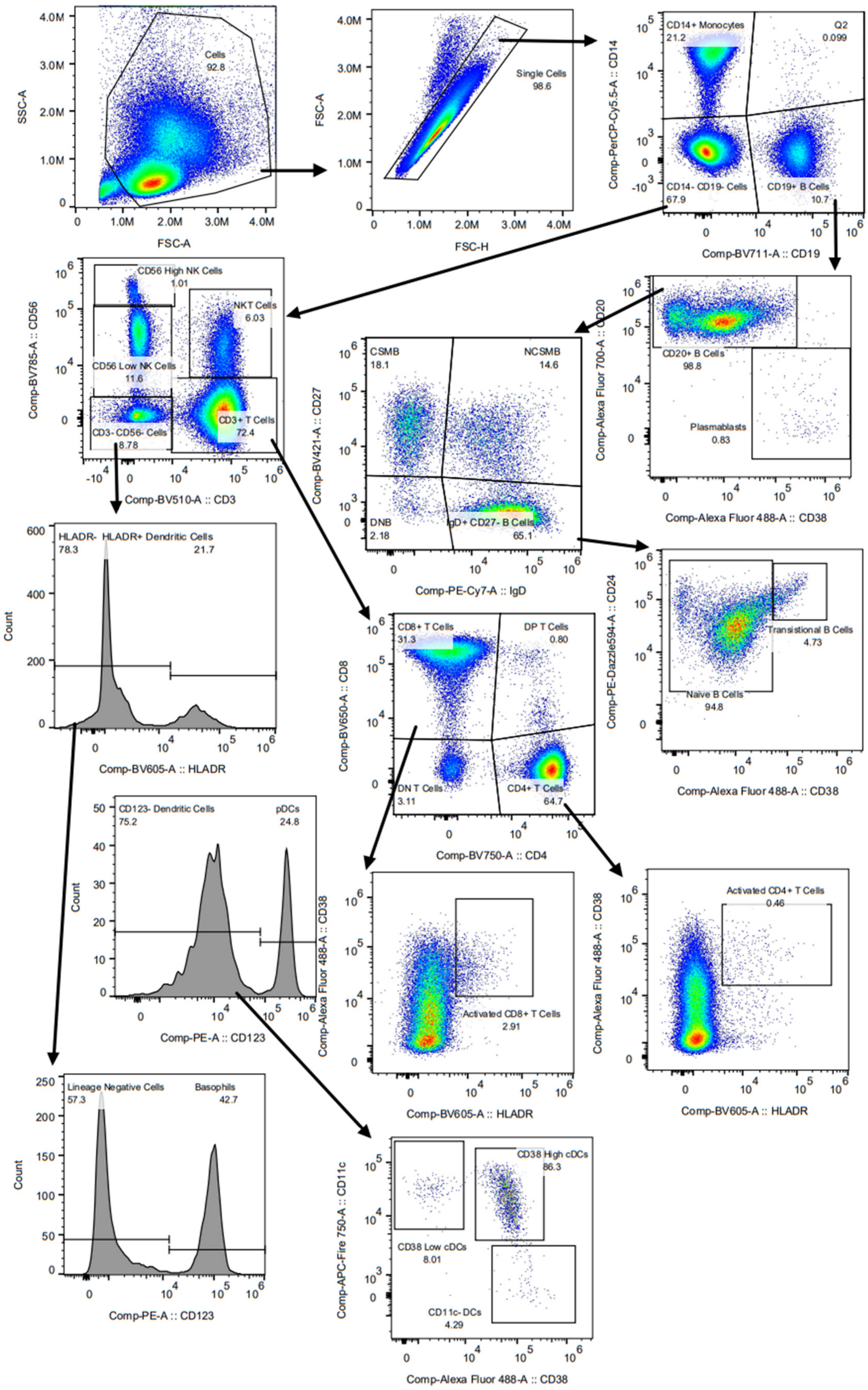

Cells
92.8
SSC-A
FSC-A
Single Cells
98.6
FSC-A
FSC-H
CD14+ Monocytes
21.2
Q2
0.099
CD14- CD19- Cells
67.9
CD19+ B Cells
10.7
Comp-PerCP-Cy5.5-A :: CD14
Comp-BV711-A :: CD19
CD56 High NK Cells
1.01
NKT Cells
6.03
CD56 Low NK Cells
11.6
CD3- CD56- Cells
8.78
CD3+ T Cells
72.4
Comp-BV785-A :: CD56
Comp-BV510-A :: CD3
CD20+ B Cells
98.8
Plasmablasts
0.83
Comp-Alexa Fluor 700-A :: CD20
Comp-Alexa Fluor 488-A :: CD38
CSMB
18.1
NCSMB
14.6
DNB
2.18
IgD+ CD27- B Cells
65.1
Comp-BV421-A :: CD27
Comp-PE-Cy7-A :: IgD
HLADR-
78.3
HLADR+ Dendritic Cells
21.7
Count
Comp-BV605-A :: HLADR
Transitional B Cells
4.73
Naive B Cells
94.8
Comp-PE-Dazzle594-A :: CD24
Comp-Alexa Fluor 488-A :: CD38
CD8+ T Cells
31.3
DP T Cells
0.80
DN T Cells
3.11
CD4+ T Cells
64.7
Comp-BV650-A :: CD8
Comp-BV750-A :: CD4
CD123- Dendritic Cells
75.2
pDCs
24.8
Count
Comp-PE-A :: CD123
Activated CD8+ T Cells
2.91
Comp-Alexa Fluor 488-A :: CD38
Comp-BV605-A :: HLADR
Activated CD4+ T Cells
0.46
Comp-Alexa Fluor 488-A :: CD38
Comp-BV605-A :: HLADR
Lineage Negative Cells
57.3
Basophils
42.7
Count
Comp-PE-A :: CD123
CD38 High cDCs
86.3
CD38 Low cDCs
8.01
CD11c- DCs
4.29
Comp-APC-Fire 750-A :: CD11c
Comp-Alexa Fluor 488-A :: CD38

**Supplementary Figure S5: Gating strategy for PBMC flow cytometry experiments.** Gating strategy used to identify major PBMC subsets. FSC-A = Forward Scatter-Area, FSC-H = Forward Scatter-Height, SSC-A = Side Scatter-Area. DNB = Double Negative B Cells. pDCs = Plasmacytoid Dendritic Cells. DN T cells = Double Negative T Cells. DP T Cells = Double Positive T Cells. CSMB = Class Switched Memory B Cells. NCSMB = Non-Class Switched Memory B Cells. Antibody panel information and cell type specific markers are described in Supplementary Tables S1 and S2, respectively.

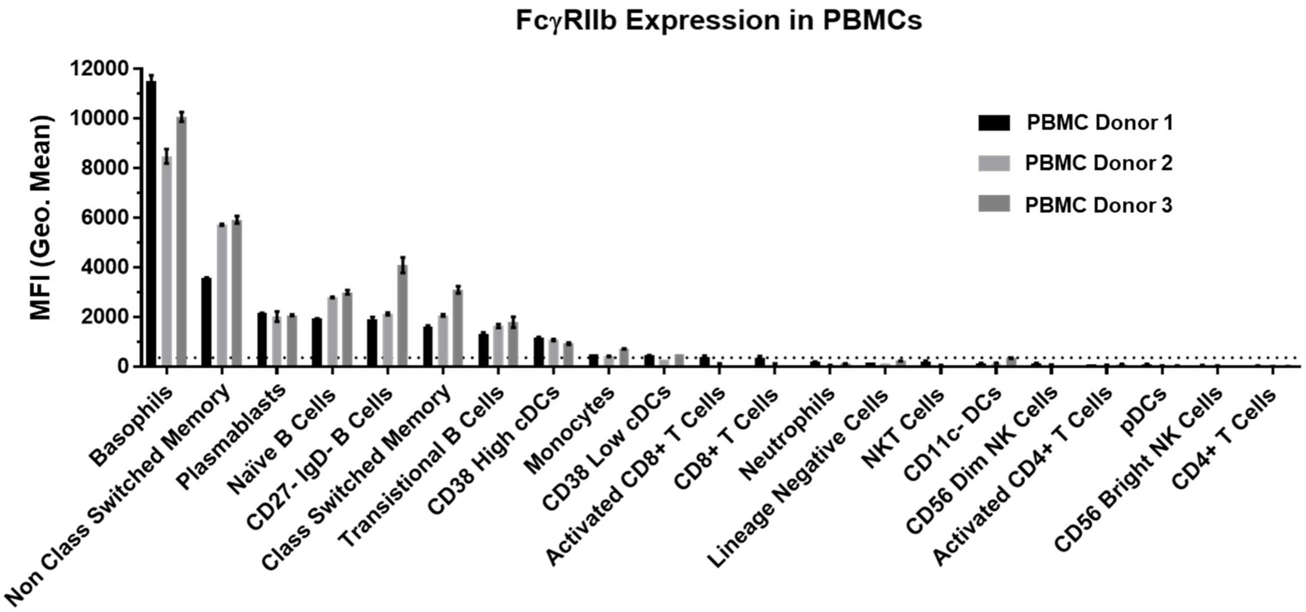


**Supplementary Figure S6: FcγRIIb expression in human PBMCs from healthy donors.** FcγRIIb expression from healthy human donors analyzed using flow cytometry with the Anti-FcγRIIb/c antibody Clone 4F5. Each donor was analyzed with $n$=2 technical replicates, with a total of $n$ = 3 PBMC donors analyzed. Dotted line represents the MFI of the APC-labeled Mouse IgG2a isotype control antibody.

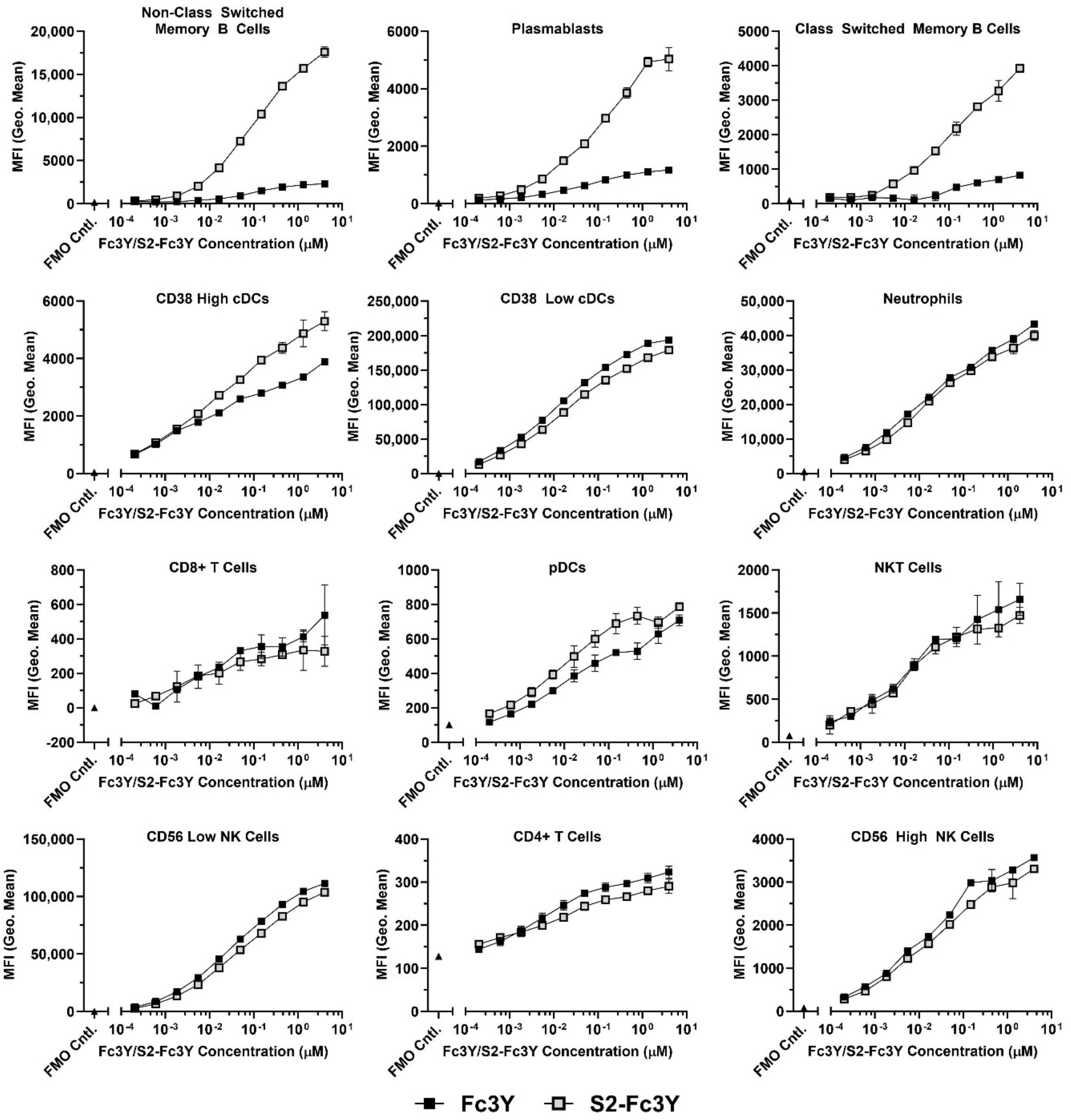


**Supplementary Figure S7: Dose response staining of human PBMCs with VivoTag 645 labeled Fc3Y and S2-Fc3Y.** Extended dose responses of Fc3Y and S2-Fc3Y to major PBMC subsets identified using the antibody panel described in Supplementary Tables S1 and S2, and the gating strategy is Supplementary Figure S5. Results are reported in the geometric mean fluorescence intensity (MFI – Geo. Mean) of VivoTag 645. Results for one donor is shown with *n* = 2 technical replicates. Data is representative of *n* = 3 PBMC donors analyzed.

**Supplemental Tables:**

| Antibody Staining Panel for PBMC Flow Cytometry | | | | | |
|---|---|---|---|---|---|
| **Antibody** | **Label** | **Antibody Clone** | **Vendor** | **Catalog #** | **Fold Dilution** |
| Anti-CD3 | Brilliant Violet 510 | OKT3 | BioLegend | 317332 | 100 |
| Anti-CD4 | Brilliant Violet 750 | SK3 | BioLegend | 344644 | 500 |
| Anti-CD8 | Brilliant Violet 650 | SK1 | BioLegend | 344730 | 250 |
| Anti-CD14 | PerCP-Cy5.5 | HCD14 | BioLegend | 325622 | 100 |
| Anti-CD19 | Brilliant Violet 711 | HIB19 | BioLegend | 302246 | 100 |
| Anti-CD20 | Alexa Fluor 700 | 2H7 | BioLegend | 302322 | 300 |
| Anti-CD24 | PE-Dazzle 594 | ML5 | BioLegend | 311134 | 100 |
| Anti-CD27 | Brilliant Violet 421 | M-T271 | BioLegend | 356418 | 100 |
| Anti-CD123 | PE | 6H6 | BioLegend | 306006 | 100 |
| Anti-CD56 | Brilliant Violet 785 | 5.1H11 | BioLegend | 362550 | 100 |
| Anti-IgD | PE-Cy7 | IA6-2 | BioLegend | 348210 | 300 |
| Anti-HLA-DR | Brilliant Violet 605 | L243 | BioLegend | 307640 | 100 |
| Anti-CD11c | APC Fire 750 | Bu15 | BioLegend | 337240 | 100 |
| Anti-CD38 | Alexa Fluor 488 | HB7 | BioLegend | 356634 | 100 |
| Anti-FcγRIIb/c | APC | 4F5 | BioLegend | 398406 | 100 |

**Supplementary Table S1: Antibody panel used for PBMC flow cytometry experiments.** All antibodies were purchased from BioLegend. Label refers to the specific fluorophore conjugated to the antibody. Fold Dilution refers to the dilution of the antibody stock into a 100μL final staining volume.

| PBMC Flow Cytometry Gating Strategy | |
|---|---|
| **Cell Type** | **Gating Strategy** |
| CD19+ B Cells | CD19+, CD14- |
| CD14+ Monocytes | CD14+, CD19- |
| CD20+ B Cells | CD19+, CD14-, CD20+ |
| Plasmablasts | CD19+, CD14-, CD20 low, CD38+ |
| Naïve B-Cells | CD19+, CD14-, CD20+, IgD+, CD27- |
| Class Switched Memory B Cells (CSMB) | CD19+, CD14-, CD20+, IgD-, CD27+ |
| Non-Class Switched Memory B Cells (NCSMB) | CD19+, CD14-, CD20+, IgD+, CD27+ |
| Double Negative B-Cells | CD19+, CD14-, CD20+, IgD-, CD27- |
| Transitional B Cells | CD19+, CD14-, CD20+, IgD+, CD27-, CD24 High, CD38 High |
| CD3+ T Cells | CD19-, CD14-, CD3+, CD56- |
| CD8+ T Cells | CD19-, CD14-, CD3+, CD56-, CD8+, CD4- |
| Activated CD8+ T Cells | CD19-, CD14-, CD3+, CD56-, CD8+, CD4-, HLA-DR+, CD38+ |
| CD4+ T Cells | CD19-, CD14-, CD3+, CD56-, CD8-, CD4+ |
| Activated CD4+ T Cells | CD19-, CD14-, CD3+, CD56-, CD8-, CD4+, HLA-DR+, CD38+ |
| Double Positive T Cells | CD19-, CD14-, CD3+, CD56-, CD8+, CD4+ |
| Double Negative T Cells | CD19-, CD14-, CD3+, CD56-, CD8-, CD4- |
| CD56 Dim NK Cells | CD19-, CD14-, CD3-, CD56 low |
| CD56 Bright NK Cells | CD19-, CD14-, CD3-, CD56 high |
| NKT Cells | CD19-, CD14-, CD3+, CD56+ |
| HLA-DR+ Dendritic Cells | CD19-, CD14-, CD3-, CD56-, HLA-DR+ |
| pDCs | CD19-, CD14-, CD3-, CD56-, HLA-DR+, CD123+ |
| CD11c- DCs | CD19-, CD14-, CD3-, CD56-, HLA-DR+, CD123-, CD38+, CD11c- |
| CD38 High cDCs | CD19-, CD14-, CD3-, CD56-, HLA-DR+, CD123-, CD38+, CD11c+ |
| CD38 Low cDCs | CD19-, CD14-, CD3-, CD56-, HLA-DR+, CD123-, CD38 low, CD11c+ |
| Basophils | CD19-, CD14-, CD3-, CD56-, HLA-DR-, CD123+ |
| Lineage Negative Cells | CD19-, CD14-, CD3-, CD56-, HLA-DR-, CD123- |

**Supplementary Table S2: Gating strategy and cell type markers for PBMC flow cytometry experiments.** "Gating Strategy" column refers to the specific marker expression for each cell type. "+" refers to positive expression, "-" refers to absence of expression. Where noted, "high" and "low" correspond to marker expression where populations express varying levels of a given marker. Abbreviations: pDCs = Plasmacytoid Dendritic Cells, DCs = Dendritic Cells, cDCs = Conventional Dendritic Cells.
